# Inflammatory Bowel Disease Types Differ in Markers of Inflammation, Gut Barrier and in Specific Anti-Bacterial Response

**DOI:** 10.3390/cells8070719

**Published:** 2019-07-13

**Authors:** Stepan Coufal, Natalie Galanova, Lukas Bajer, Zuzana Gajdarova, Dagmar Schierova, Zuzana Jiraskova Zakostelska, Klara Kostovcikova, Zuzana Jackova, Zuzana Stehlikova, Pavel Drastich, Helena Tlaskalova-Hogenova, Miloslav Kverka

**Affiliations:** 1 Institute of Microbiology of the Czech Academy of Sciences, Videnska 1083, 142 20 Prague, Czech Republic; 2 Institute for Clinical and Experimental Medicine, Videnska 1958/9, 140 21 Prague, Czech Republic; 3 Institute of Animal Physiology and Genetics of the Czech Academy of Sciences, Videnska 1083, 142 20 Prague, Czech Republic; 4 Institute of Experimental Medicine of the Czech Academy of Sciences, Videnska 1083, 142 20 Prague, Czech Republic

**Keywords:** inflammatory bowel disease, biomarkers, gut barrier, microbiota, antibodies, T cells

## Abstract

Crohn’s disease (CD), ulcerative colitis (UC) and inflammatory bowel disease (IBD) associated with primary sclerosing cholangitis (PSC-IBD), share three major pathogenetic mechanisms of inflammatory bowel disease (IBD)-gut dysbiosis, gut barrier failure and immune system dysregulation. While clinical differences among them are well known, the underlying mechanisms are less explored. To gain an insight into the IBD pathogenesis and to find a specific biomarker pattern for each of them, we used protein array, ELISA and flow cytometry to analyze serum biomarkers and specific anti-microbial B and T cell responses to the gut commensals. We found that decrease in matrix metalloproteinase (MMP)-9 and increase in MMP-14 are the strongest factors discriminating IBD patients from healthy subjects and that PSC-IBD patients have higher levels of Mannan-binding lectin, tissue inhibitor of metalloproteinases 1 (TIMP-1), CD14 and osteoprotegerin than patients with UC. Moreover, we found that low transforming growth factor-β1 (TGF-β1) is associated with disease relapse and low osteoprotegerin with anti-tumor necrosis factor-alpha (TNF-α) therapy. Patients with CD have significantly decreased antibody and increased T cell response mainly to genera *Eubacterium*, *Faecalibacterium* and *Bacteroides*. These results stress the importance of the gut barrier function and immune response to commensal bacteria and point at the specific differences in pathogenesis of PSC-IBD, UC and CD.

## 1. Introduction

Inflammatory bowel diseases (IBD), i.e., Crohn’s disease (CD) and ulcerative colitis (UC), are severe chronic inflammatory illnesses of the gastrointestinal tract, affecting more than 0.3% of the people in many countries [1]. Although their etiology and pathogenesis is not fully understood, it is generally accepted that the inflammation results from an aberrant immune response to antigens of resident gut microbiota in genetically susceptible individuals [2]. Moreover, primary sclerosing cholangitis (PSC), chronic liver disorder characterized by inflammation and stenosis of the bile ducts, with concomitant IBD (PSC-IBD) has recently emerged as another form of IBD [3]. Despite the well-established headlines in IBD therapy, discontinuation of pharmacological intervention due to the inefficiency or adverse events is still common in all types of IBD therapy [4,5,6]. Ability to predict the disease relapses and complications or suggest the ideal therapy for a particular patient during the time of diagnosis is a worthy goal of IBD diagnostics. Taking into account the complex and intertwined pathogenesis together with distinct forms of IBD, studies of those mechanisms may yield suitable biomarkers. Three major mechanisms are involved in IBD pathogenesis, gut microbiota dysbiosis, gut barrier failure and dysregulation of the immune system.

In humans, gut microbiota represents a complex ecosystem that consists of more than 1000 species of bacteria, five genera of Archaea, 66 genera of fungi and an ill-defined number of viruses, mostly bacteriophages [7,8,9]. The gnotobiotic (i.e., germ-free or artificially colonized animals) studies clearly showed that without this complex ecosystem, the immune system and many other physiological functions would never reach their full potential [10]. While gut microbiota cannot induce intestinal inflammation on its own [11], imbalances in intestinal microbiota (i.e., dysbiosis), or the presence of commensal bacteria with increased virulence in IBD patients, could cause excessive anti-microbial immune response [12,13,14,15]. It is still unclear, however, if these microbial perturbances in IBD are cause, consequence or just a confounding factor [16].

The gut barrier is a complex apparatus consisting of a mucus layer, a tightly connected epithelium supported by mucosal immune cells and their products that protect an organism’s integrity [17]. Disruption of this barrier (defects of the epithelial continuity) increases its permeability allowing the excessive contact of the luminal antigens with the immune cells, which is one of the key steps in pathogenesis of IBD and several other diseases [18]. The gut barrier disruption is responsible for many IBD symptoms even during the mucosal healing [19]. Both UC and CD patients with an active disease have severe impairment of this barrier on multiple levels [20,21]. Several noninvasive biomarkers of gut barrier failure were suggested. Since both Intestinal and Liver Fatty Acid-Binding Proteins (I-FABP, L-FABP) reflect gut epithelium damage, they were previously successfully used as early biomarkers for severe neonatal emergencies, such as necrotizing enterocolitis or gastroschisis [22,23,24]. Because the matrix metalloproteinase (MMP) system has an important role in the gut barrier remodeling, both fecal and serum MMP-9 levels were suggested as promising biomarkers of gut barrier health [25,26].

The impairment of host–microbe interactions in IBD pathogenesis is supported by genome-wide association studies, which identified an association of IBD with multiple polymorphisms in genes encoding regulation of immune processes including the recognition, processing and killing of microorganisms [27,28]. Disruption of regulatory T cell functions and impairment of the mucosal immune response to normal microbiota play a crucial role in the pathogenesis of chronic intestinal inflammation [29]. Although the typical T helper (Th)1 and Th17 response is associated with CD pathogenesis, the presence of antibodies to some microbial constituents in sera of patients suggests a much broader spectrum of immune reactions in IBD [30].

The diagnosis of IBD and its clinical staging is still based mainly on the patient’s history and medical examination, where endoscopy plays a major part. Several serological tests were proposed to improve the IBD diagnostics and some of them showed promising predictive value. The anti-Saccharomyces cerevisiae antibodies (ASCAs) reacting to the mannan protein in the *Saccharomyces cerevisiae* are significantly increased and highly specific for CD patients even if they have clinical remission and the perinuclear anti-neutrophil cytoplasmic antibodies (pANCAs) are increased in UC patients [31,32,33]. Apart from ASCA, serum antibodies to other microbial antigens were not only a source of potential biomarkers for IBD diagnosis and differential diagnosis, but also suggested the importance of anti-microbial response in IBD pathogenesis. These biomarkers included *Escherichia coli* outer membrane porin C (anti-OmpC), anti-flagellin (anti-Cbir1) [34] and the anti-I2 component of *Pseudomonas fluorescens* (anti-I2) [35]. Other biomarkers, such as serum and fecal calprotectin, fecal lactoferrin, S100A12, Lipocalin-2, showed promising results in relapse prediction. However, the utility of these markers in daily clinical practice is still rather low [36,37,38].

In this study, we performed broad analysis of serum and peripheral blood mononuclear cells (PBMCs) biomarkers, including chemokines, cytokines, specific antibodies and specific anti-microbial T cell reactivity to gain an insight into the IBD pathogenesis and to find biomarker pattern specific for each form of IBD.

## 2. Materials and Methods

### 2.1. Study Population

All individuals were recruited from the patients admitted to the Hepatogastroenterology Department of the Institute for Clinical and Experimental Medicine (IKEM), Prague, Czech Republic, between May 2015 and December 2018. In total, we enrolled 119 patients with different forms of IBD; CD, UC and PSC-IBD and 28 healthy individuals served as controls (HC) (Table 1). Since CD can involve different parts of gastrointestinal tract, all IBD patients had colonic involvement to minimize the variability. Serum was aliquoted and stored at −20 °C until analyses.

### 2.2. Antibody Array Assay for Serum Biomarkers

A training set of 18 samples, six each of HC, UC and CD, was assayed for the relative amount of 507 human proteins using RayBio Label-Based (L-Series) Human Antibody Array L-507 according to the manufacturer’s protocol (RayBiotech, Peachtree Corners, GA, USA). The target proteins included cytokines, chemokines, adipokines, growth factors, angiogenic factors, proteases, soluble receptors and soluble adhesion molecules. The signals were scanned at a wavelength of 532 nm using GeneTAC UC4 Microarray Scanner (Genomic Solution, United Kingdom; resolution, 10 µm), and the resulting image was analyzed and processed in AG Scan software (ver. 18.7. 2007, The GenoToul bioinformatics, France) [39]. To compare the median fluorescence intensity (MFI) values, we subtracted the background staining and normalized the data to the positive control MFI average for all arrays, and then transformed to z-scores for each protein. The classifiers for HC, UC and CD were analyzed by nearest shrunken centroid method by Prediction Analysis of Microarrays (PAM; ver. 1.56) package for R (ver. 3.5.2; R Foundation for Statistical Computing, Vienna, Austria) [40].

### 2.3. ELISA for Serum Biomarkers

Next, we selected several biomarkers found by microarray profiling and several other, proposed markers and quantified them in the serum by ELISA (Table 2). Due to the limited amount of sample, not all samples were analyzed for all biomarkers.

### 2.4. Bacterial Antigen Preparation

We selected typical representatives of healthy Czech gut microbiota, using data from our previous study [41]. Different bacteria were cultured in their respective optimal media for 24 h at 37 °C (Table S1). Fresh bacterial culture was centrifuged, pellets were washed in sterile water (B. Braun Medical, Prague, Czech Republic) and inactivated by French press (French Pressure Cell Press Model FA-078, SLM Instruments) at 1500 psig, the pressurizing procedure was repeated three times. Samples were freeze dried in a lyophilizer (Lyovac GT 2, Leybold Heraeus) and stored in aliquots at -28 °C until analyses. The following bacterial strains were used for PBMCs stimulation or for antigen coating in indirect ELISA: *Lactobacillus plantarum*, *Bifidobacterium adolescentis*, *Blautia coccoides*, *Roseburia intestinalis*, *Eubacterium rectale*, *Faecalibacterium prausnitzii*, *Ruminococcus flavefaciens*, *Bacteroides thetaiotaomicron*, *Prevotella ruminicola* and *Escherichia coli*.

### 2.5. Peripheral Blood Mononuclear Cells (PBMC)

Human Peripheral Blood Mononuclear Cells (PBMCs) were isolated by Ficoll-Paque Plus (GE-Healthcare; Cat# 17-1440-03) density gradient centrifugation (740 × *g*, 30 min, room temperature (RT), brake OFF) from heparinized blood and stored at −150 °C using freeze-thaw method optimized for maximum viability [42]. Briefly, after the collection the cells were washed in sterile pre-warmed phosphate-buffered saline (PBS) (330 × *g*, 10 min, RT), re-suspended in pre-warmed Roswell Park Memorial Institute (RPMI) medium (Sigma-Aldrich; Cat# R0883), counted and re-suspended at 20 × 10^6^ /mL live cells in RPMI containing 60% Fetal calf serum (FCS; Biochrom GmbH, Germany; Cat# S 0115). Next, the equal volume of pre-warmed mixture of 80% FCS and 20% DMSO (Sigma-Aldrich; Cat# D 2650) was gently added drop wise with gentle shaking after each drop to equalize the cryopreservant. After the 5 min incubation (RT), cells were aliquoted to cryovials and gently frozen in a Mr. Frosty Freezing Container (Thermo Fisher Scientific; Cat# 5100-0001) at −80 °C. After 12–48 h, cells were transferred to −150 °C for long term storage until analyses. For thawing cryotubes were placed in a 37 °C water bath for 8 min, then transferred to a 15 mL tube, diluted drop wise with 8 mL of pre-warmed RPMI medium, centrifuged (300 × *g*, 5 min, RT) and the supernatant was discarded. After another washing step, the cells were counted using Trypan blue exclusion and diluted to 2 × 10^6^ /mL live cells. Cells were then transferred to sterile 96U-well tissue culture plate (TPP, Trasadingen, Switzerland; Cat# 92197) at 100 µL/well in complete RPMI medium containing 10% FCS, 1% antibiotic-antimycotic solution (Sigma-Aldrich; Cat# P 0781) and 1% L-glutamine solution (Sigma-Aldrich; Cat# 1.00289) and placed into the humidified incubator (37 °C, 5% CO_2_) for 2 h before the stimulation. Next, 100 µL/well of the stimulus was added and the cells were incubated under similar conditions for another 14 h. The final concentration of microbial lysate was 10 µg/mL and 1 µg/mL of *Staphylococcus aureus* toxin B (SEB; Sigma-Aldrich; Cat# S 4881) served as a positive control.

### 2.6. Indirect Enzyme-Linked Immunosorbent Assays (ELISA)

The serum concentrations of anti-bacterial antibodies in Immunoglobulin M (IgM), Immunoglobulin G (IgG) and Immunoglobulin A (IgA) isotypes were analyzed by in-house developed indirect ELISA. Bacterial lysates, were dissolved in phosphate buffered saline (PBS) and incubated at 0.1 mg/mL (*Prevotella*, *Ruminococcus* and *Bacteroides*), 0.5 mg/mL (*Faecalibacterium*) or 1 mg/mL overnight in the 96F-well plate (NUNC Maxisorp; Cat# 442404). Optimal concentration of the coated lysates was extensively tested with sera of HC and IBD patients, but there were no major differences between 0.1 and 5 mg/mL for most lysates. Next, plates were washed with 1xPBS containing 0.05% Tween^®^ 20 (Merck KGaA, Darmstadt, Germany). Each well was blocked with 1% Bovine Serum Albumin (BSA; Merck) for 1 h. After the washing procedure, patient serum samples were applied in appropriate dilution. After 2 h of incubation plates were washed and corresponding secondary antibody (Peroxidase-conjugated AffinniPure F(ab’)2 fragment goat anti-human Fc fragment specific; Jackson ImmunoResearch Laboratories, Inc., Ely, UK; Cat# 109-036-170, 109-036-011 or 109-036-129) was added and incubated 1 h in the dark. After washing, substrate solution was added and plates were incubated for 5 min in the dark. Absorbance was measured at 450 nm and 650 nm by spectrophotometer (Multiskan Ascent Plate Reader 96/384, MTX Lab Systems). Selected serum sample was used on all plates to serve as a standard. Its serial dilutions were used for the antibody response quantification with optical density (OD) at 1:200 defined as 1000 arbitrary units (AU).

### 2.7. Flow Cytometry Analysis (FACS)

Cells were stained with the Fixable Viability Dye eFlour 780 (eBioscience, San Diego, CA, USA; Cat# 65-0865-18) and following fluorescently labeled monoclonal antibodies: Fluorescein isothiocyanate (FITC) anti-human CD3 Antibody (UCHT1; Biolegend, San Diego, CA, USA; Cat# 300452), Qdot 605 anti-human CD4 Antibody (S3.5; Invitrogen, Carlsbad, CA, USA; Cat# Q 1008), Alexa Flour 700 anti-human CD8 Antibody (SK1; Biolegend; Cat# 344724), Brilliant Violet 711 anti-human Interleukin (IL)-17A Antibody (BL168; Biolegend; Cat# 521327), Allophycocyanin (APC) anti-human IL-4 Antibody (8DE-8; eBioscience, Cat# 17-7049-81), Brilliant Violet 510 anti-human Tumor necrosis factor-alpha (TNF-α) Antibody (Biolegend; Cat# 502949), Phycoerythrin (PE) anti-human Interferon-gamma (IFN-γ) Antibody (4S.B3; eBioscience; Cat# 12-7319-81), PE-Cyanine7 IL-2 Antibody (MQ1-17H12; eBioscience; Cat# 25-7029-41), Brilliant Violet 421 anti-human CD154 (24-31; Biolegend; Cat# 310 824).

For intracellular cytokine staining, cells were stimulated for 14 h with Staphylococcal enterotoxin B from *Staphylococcus aureus* (final concentration 1 µg/mL; Merck) or with corresponding bacterial lysates (final concentration 10 µg/mL). Brefeldin A (final concentration 3 µg/mL, eBioscience) and Monensin (final concentration 2 µM, eBioscience) were added and after 4 h the cells were stained with Fixable Viability Dye, fixed with Intracellular (IC) Fixation Buffer (Invitrogen), and stained for cytokines in Permeabilization Buffer (Invitrogen). Gating on CD154 (CD40 ligand) was combined with intracellular cytokine analysis to focus on activated memory T helper cells as described previously [43].

Human Fc-γ receptor (FcR) Binding Inhibitor Purified (eBioscience) was used for inhibition of the non-specific FcR-mediated binding of monoclonal antibodies. UltraComp eBeads Compensation Beads (Invitrogen) were used for compensations. Cells were analyzed on FACS LSR II (BD Biosciences, San Jose, CA, USA). Data were analyzed with FlowJo (version 7.2.5., Tree Star, Inc., Ashland, OR, USA).

### 2.8. Ethics Statement

All subjects gave their informed consent for inclusion before they participated in the study. The study was conducted in accordance with the Declaration of Helsinki, and the protocol was approved by the Ethics Committee of Institute of Clinical and Experimental Medicine and Thomayer Hospital (G 14-08-45).

### 2.9. Statistical Analysis

Non-parametric Kruskal–Wallis test with Dunn’s multiple comparison test was used to compare multiple experimental groups and the Mann–Whitney test was used to compare two experimental groups. Non-parametric paired Friedman test with Dunn’s multiple comparison test was used to compare the CD154 expression after the cultivation with different microbial antigens with CD154 expression in non-stimulated sample. The data are presented as the median ±95% confidence interval and differences were considered statistically significant at *p* ≤ 0.05. GraphPad Prism statistical software (version 8.1.1, GraphPad Software, San Diego, CA, USA) was used for analyses.

Regression analysis was performed in R and the effect of each biomarker on Akaike information criterion (AIC) was determined in the nnet package (ver. 7.3-12). Next, we performed both backward elimination and forward selection based on AIC to determine the best regression model to discriminate between the two states. The composite receiver operating characteristic (ROC) curves were constructed and their area under the curve (AUC) was calculated using ROCR package (ver. 1.0-7).

Hierarchical clustering was performed using the Unweighted Pair Group Method with Arithmetic Mean (UPGMA) and the heatmap.plus (ver. 1.3) package for R.

## 3. Results

### 3.1. Healthy Subjects, CD and UC Patients Each Have a Distinct Cytokine Signature in Human Serum

**Figure 1:**
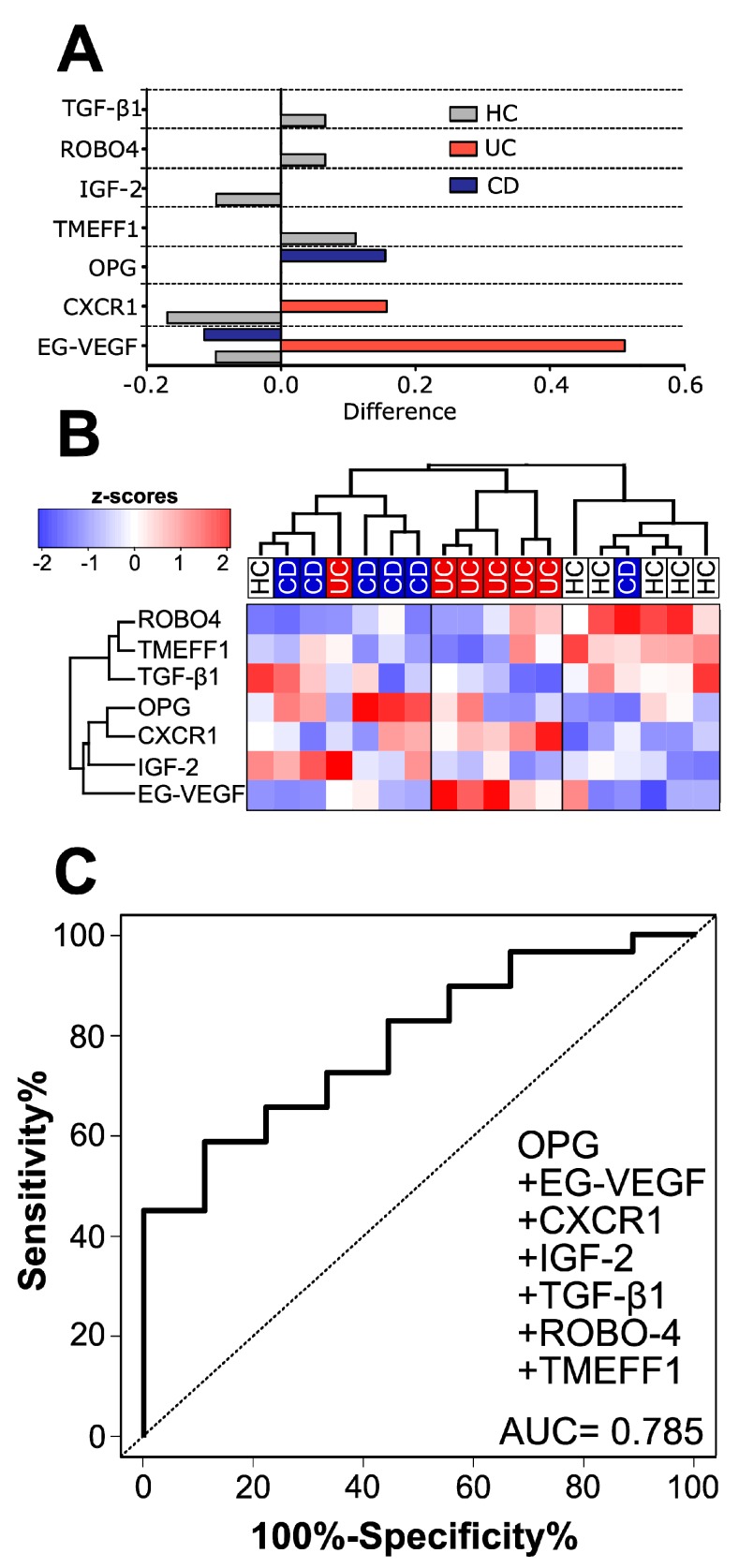
HC can be easily distinguished from IBD by only seven proteins, but separation of UC and CD is not as clear: (**A**) Shrunken differences for the seven differently abundant proteins in sera; (**B**) Heat map and cluster analysis of the chosen proteins. “HC” healthy controls, “UC” ulcerative colitis, “CD” Crohn’s disease.; (**C**) Composite receiver operating characteristic (ROC) curve for the seven proteins analyzed by ELISA with the training set of samples HC (*n* = 10) and IBD patients consisting of UC (*n* = 9), CD (*n* = 10) and PSC-IBD (*n* = 10).

Using the nearest shrunken centroid method, we searched for typical patterns of serum proteins that could differentiate between HC, CD and UC (Figure 1A). Subsequent cluster analysis of these proteins showed good separation of HC from IBD patients and their separation between UC and CD (Figure 1B). When the training set of patients (HC = 9, UC = 9 and CD = 10) was analyzed by ELISA, the ability to classify was only marginal, with osteoprotegerin (OPG) having the strongest effect. Both tomoregulin 1 (TMEFF1) and roundabout guidance receptor 4 (ROBO4) had only a negligible role on the HC vs. IBD classification (AUC = 0.785) and OPG was the strongest discriminating factor. In these cases, ELISA was in agreement with the protein array, with OPG being increased in CD and endocrine-gland-derived vascular endothelial growth factor (EG-VEGF) in UC, but neither difference was statistically significant.

### 3.2. Validation of Microarray Data by ELISA

**Figure 2:**
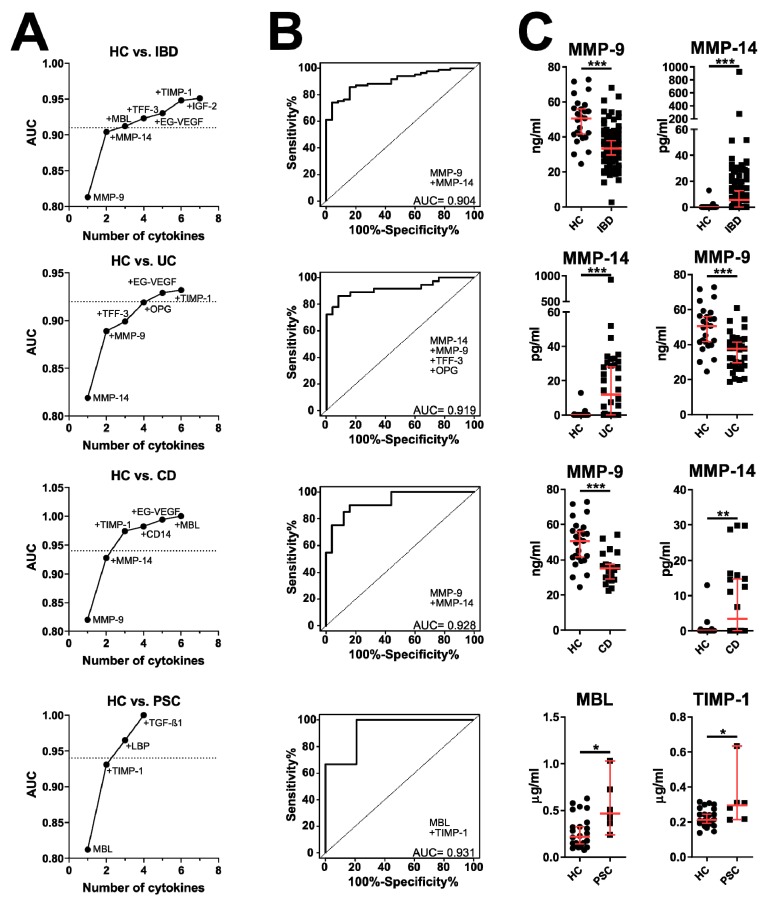
Cytokine patterns discriminating different forms of IBD from healthy controls. (**A**) Relative importance for each cytokine for the AUC increment within the best model found by regression analysis and (**B**) composite ROC curve analysis with the reliable discriminating power (AUC > 0.9). (**C**) Quantitative plot of the two most efficient discriminating factors analyzed by Mann Whitney test. * *p* < 0.05, ** *p* < 0.01, *** *p* < 0.001. Full quantitative comparison across all types of IBD is in Figure S1. Healthy controls (HC, *n* = 25), IBD patients (*n* = 85), UC patients (*n* = 36), CD patients (*n* = 20), PSC patients without concomitant IBD (PSC; *n* = 6).

**Figure 3:**
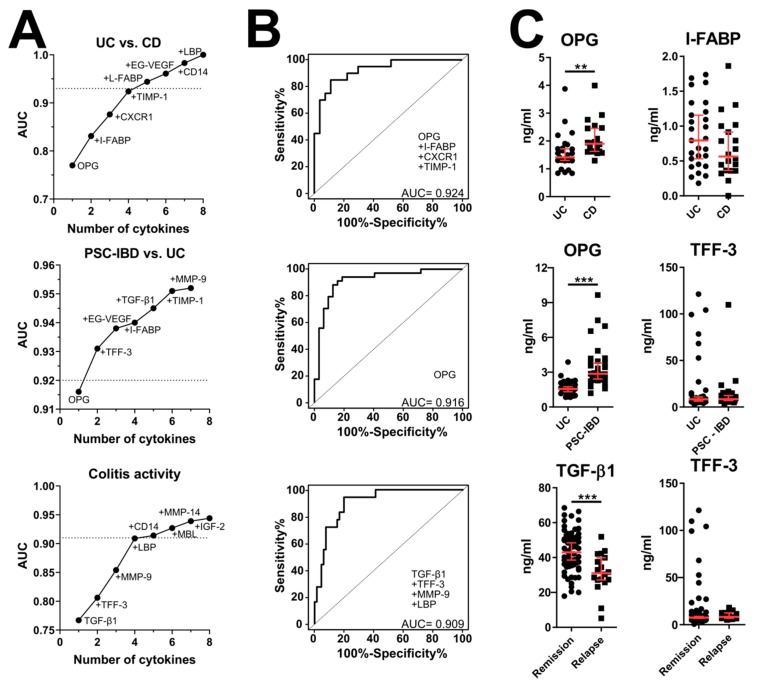
Significant differences in serum biomarkers between different types of IBD and activity. (**A**) Relative importance for each cytokine for the AUC increment within the best model found by regression analysis and (**B**) composite ROC curve analysis with the reliable discriminating power (AUC > 0.9). (**C**) Quantitative plot of the two most efficient discriminating factors analyzed by Mann–Whitney test. ** *p* < 0.01, *** *p* < 0.001. HC (*n* = 25), UC patients (*n* = 36), CD patients (*n* = 20), PSC-IBD patients (*n* = 32), Remission (*n* = 66), Relapse (*n* = 18).

Next, we analyzed the predictive value of these potential serum biomarkers using ELISA. Moreover, we selected other serum biomarkers of the gut barrier (MMP-9, MMP-14, tissue inhibitor of metalloproteinases 1 (TIMP-1), zonulin, I-FABP, L-FABP and trefoil factor 3 (TFF-3)) or inflammation (mannan-binding lectin (MBL), CD14, lipopolysaccharide-binding protein (LBP), EndoCab, serum amyloid A (SAA), D-amino-acid oxidase (DAAO) and TNF receptor superfamily member 19 (TNFRSF19) that may be involved as well. This analysis was performed in a larger cohort of patients including patients with UC, CD and PSC-IBD. We focused on the analysis of disease type, presence of the complications, disease activity, extent and localization. Except for some patient’s samples, both interleukin-8 receptor, alpha (CXCR1) and TNFRSF19 were below the detection limit of 195 ng/mL and 781 pg/mL, respectively. First, we found that many of these biomarkers are significantly increased in PSC-IBD patients. Except for the significant increase in TIMP-1 in CD, none of the other groups differed significantly from healthy controls (Figure S1), but the proteins from the MMP system (MMP-9, MMP-14 and TIMP-1) were the strongest discriminating factor in all forms of IBD (Figure 2B,C). Decreased serum concentration of OPG is typical for UC, as compared to CD and PSC-IBD (Figure 3), but while LBP was the strongest predictor of them all (AUC = 0.663), neither serum biomarker was capable to predict extent of colitis well (Figure S2).

### 3.3. Serum Antibodies Against Bacteria

**Figure 4:**
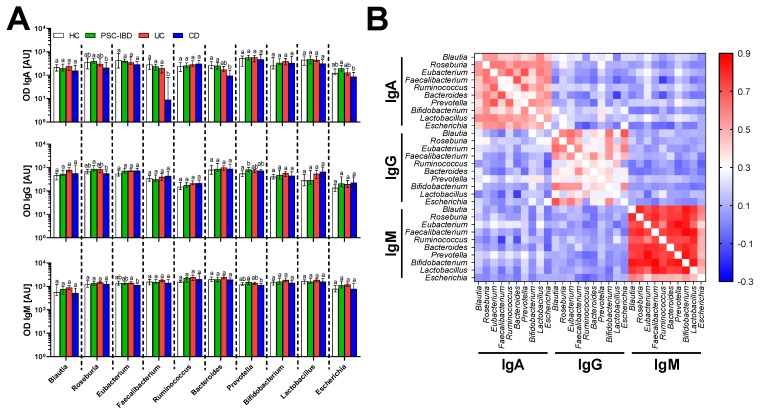
Differences in antibody response among patients with different forms of IBD and healthy controls. (**A**) Comparison of specific anti-bacterial antibody response. Different letters indicate statistically significant differences. (**B**) Correlation matrix showing Spearman’s rank correlation coefficient. HC (*n* = 27), PSC-IBD (*n* = 41), UC (*n* = 52), CD (*n* = 20).

The importance of gut barrier functions and gut microbiota for the IBD pathogenesis suggested specific antibodies to gut commensal microbiota as suitable biomarkers. Therefore, we analyzed IgA, IgG and IgM antibodies specific to gut commensal bacteria in serum of IBD patients and healthy controls. We found that patients with IBD respond in similarly to most commensal bacteria as healthy controls, with few notable exceptions (Figure 4A). CD patients have generally lower antibody response, with significantly decreased IgA response to *Faecalibacterium* and *Bacteroides* as compared to healthy controls. In fact, 23% of patients with UC and 40% of patients with CD but none with the PSC-IBD have undetectable IgA response to *Faecalibacterium*. Further analysis showed clear positive correlation within isotypes, but not across them, suggesting that each of them acts independently and that gut commensals share antigenic determinants (Figure 4B). The latter case is supported by the fact that the strongest correlation is among least specific IgM and the weakest is among the generally more specific IgG.

### 3.4. Circulating Gut Microbiota Reactive T-cells

**Figure 5:**
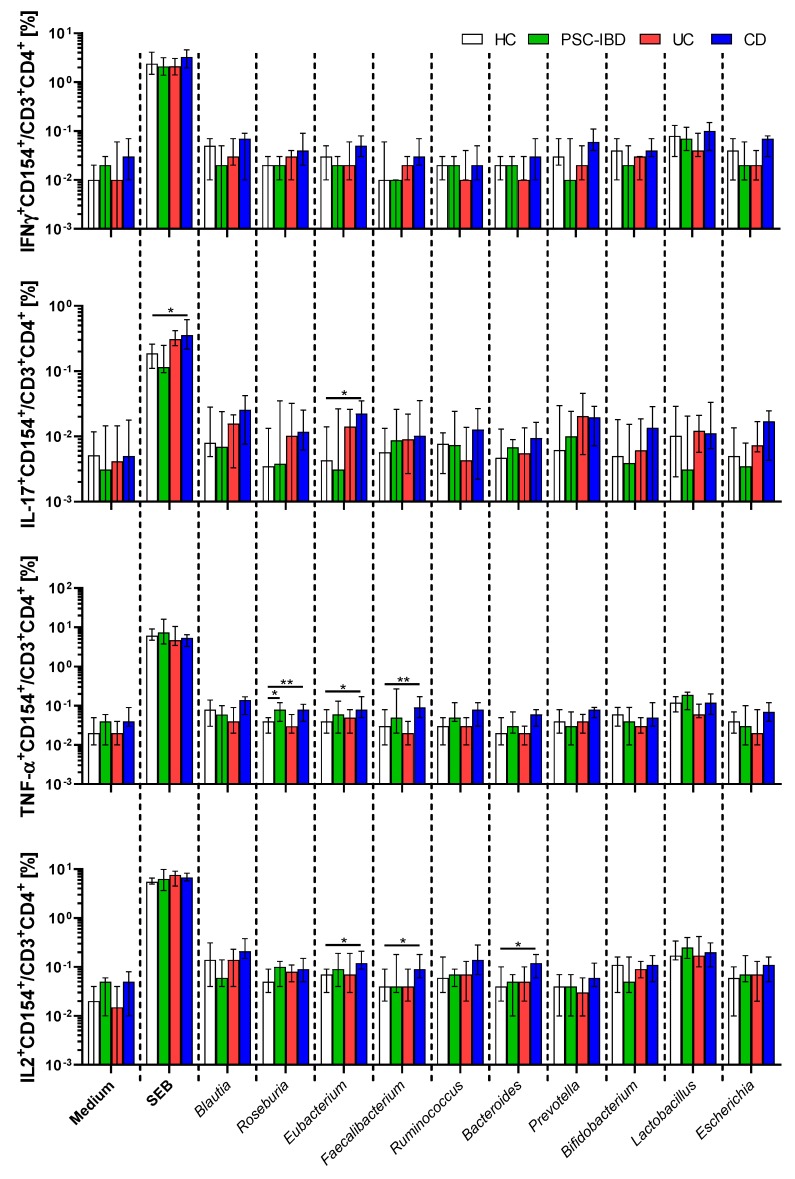
Circulating microbiota-reactive T cells react more strongly in CD patients than in any other form of IBD as analyzed by the Kruskal–Wallis test with Dunn’s multiple comparison test vs. HC group. * *p* < 0.05, ** *p* < 0.01. HC (*n* = 19), PSC-IBD (*n* = 9), UC (*n* = 15), CD (*n* = 17).

While there is some specific pattern in CD154 expression on helper T cells among healthy controls and in different forms of IBD, PBMCs from all subjects reacted strongly to antigens from Clostridiales XIVa cluster, *Prevotella*, *Lactobacillus* and *Escherichia* in all subjects regardless of presence or absence of IBD. IBD patients have this spectrum of reactivity broadened to *Bifidobacteria* in patients with UC, *Faecalibacterium* in patients with PSC-IBD and CD and *Ruminococcus* in patients with CD (Figure S6). Circulating CD4^+^CD154^+^ T cells react to gut bacteria with production of several pro-inflammatory cytokines. However, their reactivity in patients with UC and PSC-IBD is generally similar to HC. T cells from CD patients react more strongly to antigens from *Roseburia*, *Eubacterium*, *Faecalibacterium* and *Bacteroides* (Figure 5). The potential for maximum cytokine production, after super-antigen stimulation, is similar for all tested groups in all cytokines but IL-17. Upon stimulated with SEB, PBMCs from CD patients have higher significantly proportion of IL-17+CD154+ T cells than HC.

### 3.5. Effect of IBD Treatment

Next, we analyzed how the therapy influences the serum biomarkers (Figure S3), antibodies (Figure S4) and anti-microbial T cell response (Figure S5). We found that the effect of different treatments was generally milder than the effect of disease type, but there were several factors that clearly distinguished the effect of drugs. Mesalazine (5-ASA) increased MMP-14 and decreased IgM against *Bacteroides*, Azathioprine decreased the proportion of IFN-γ production by CD154^+^CD4^+^ T cells after their stimulation with *Roseburia* and oral *E. coli* Nissle 1917 (Mutaflor) increased serum IgG against *Bifidobacteria*. Most changes were, however, induced by anti-TNF-α treatment, which significantly decreased serum OPG, increased IFN-γ production from *Roseburia*- or *Escherichia*-treated CD154^+^CD4^+^ T cells and increased TNF-α production from *Escherichia* treated CD154^+^CD4^+^ T cells.

## 4. Discussion

Common pathogenetic mechanisms, gut microbiota dysbiosis, gut barrier failure and immune system dysregulation, link the different types of IBD. Yet there are clear clinical differences between CD, UC and PSC-IBD. Here, we found several serum markers that not only distinguish the major forms of IBD, but also mirror its activity or treatment. Moreover, we compared anti-microbial antibody and T cell responses to gut commensal bacteria prevalent in the Czech population, finding clear shifts in CD patients.

Using protein array, we found that out of the 507 serum proteins, high EG-VEGF and CXCR1 are strongly associated with UC and low EG-VEGF and high OPG are typical for CD. This is an interesting distinction between the two major forms of IBD, suggesting that they may differ in angiogenesis and inflammation regulation. Members of the VEGF family are not only key positive mediators of angiogenesis, but they also have a pro-inflammatory role in inflammatory diseases, including IBD [44,45,46]. While mainly linked to reproduction, EG-VEGF (Prokineticin 1) may mediate similar biological effects. In fact, human monocytes activated with EG-VEGF have elevated IL-12 and TNF-α and down-regulated IL-10 production in response to Lipopolysaccharide (LPS) [47]. This effect may decrease the triggering threshold in monocytes in the intestinal wall, thus worsening the inflammation when the gut barrier is breached in the vicinity of ulcers in patients with UC. CXCR-1 is a G protein-coupled receptor, which can recruit the neutrophils in the site of inflammation, induce their oxidative burst and degranulation, thus worsening the local inflammation [48,49]. This supports our findings since the neutrophils are major constituents of inflammatory infiltrate in UC [50]. OPG is not only a key factor in bone density regulation [51] but it also affects cell turnover, differentiation, death and survival [52]. Previous studies found elevated serum OPG in patients with IBD and showed that OPG correlated significantly with concentration of pro-inflammatory cytokines (e.g., TNF-α) suggesting that OPG production is influenced by cytokine milieu in chronic inflammation [53]. The increase in serum OPG in patients with IBD described by us and others [54] does not support the fact that IBD patients have generally worse bone mineral density than healthy controls [55]. While the negative effect of corticosteroid therapy on bone metabolism is well established [55], we did not find it mirrored in OPG levels in corticosteroid-treated patients. Nevertheless, we found a significant decrease in serum OPG in IBD patients on anti-TNF-α treatment. This may be caused by the feedback reaction of the organism to the anti-inflammatory treatment and efficient blockage of TNF-α. Moreover, this may explain the discrepancy in OPG with studies performed before the widespread use of TNF-α blockers. In our training cohort, we found OPG increased in CD, but not in UC patients. This may be due to the fact that protein array gives only relative quantification and that our UC cohort of patients for protein array consisted of only 6 patients, which may be too low. Therefore, we quantified this interesting factor with ELISA in the extended cohort of HC, UC and CD subjects. We found that the combination of OPG with six other proteins (EG-VEGF, CXCR-1, insulin-like growth factor 2 (IGF2), transforming growth factor-β1 (TGF-β1), ROBO4 and TMEFF1) can reasonably well distinguish healthy individuals from those with IBD, so we selected the strongest predictors and performed the analysis on the extended experimental set. Interestingly, OPG was not only the strongest factor distinguishing CD and UC, on its own (AUC = 0.916) it could easily distinguish UC and PSC-IBD patients, where its levels are even higher than in CD, despite the fact that all PSC-IBD patients showed UC-like features of inflammation. This suggests that inflammatory control in PSC-IBD is very different from UC. This stronger response was not limited to the OPG, because we found multiple serum biomarkers (e.g., MMP-14, MBL, CD14, I-FABP, L-FABP, ROBO4) increased in patients with PSC-IBD.

The extracellular matrix and connective tissue of the gut wall in healthy subjects is constantly remodeled and repaired by the carefully regulated release of matrix metalloproteinases (MMPs) and their inhibitors (TIMPs). The disturbance in the balance between synthesis and degradation of the extracellular matrix can result in typical features of IBD, such as ulcer formation, fibrosis or organ destruction [56]. In our dataset, decreased MMP-9 and increased MMP-14 were the strongest factors distinguishing IBD patients from healthy controls. Moreover, increased TIMP-1 was the second strongest factor distinguishing PSC patients without IBD from healthy controls and it was significantly increased in patients with PSC-IBD. However, the ability of TIMP-1 to distinguish between healthy and PSC patients may be influenced by the rarity of patients suffering from PSC without the concomitant IBD [3], so these results need to be verified on a larger cohort of patients. These findings clearly suggest the importance of the matrix metalloproteinase system in IBD pathogenesis. MMP-14 is a collagenase responsible for collagen degradation during re-modeling and for the activation of other enzymes and factors, thus triggering a proteolytic cascade or modulating important inflammatory factors [57]. The increase in MMP-14 we found in IBD patients may be a factor that mirrors the constant pathological remodeling of gut mucosa. MMP-9 is strongly expressed in inflamed mucosa during IBD [58] and several reports found increased serum MMP-9 as a marker for IBD activity in pediatric and adult IBD [59,60]. In our dataset, patients with IBD had significantly lower serum MMP-9 as compared to controls and there were neither significant differences in serum MMP-9 between patients with relapse and remission nor any correlation between MMP-9 and C-reactive protein (CRP). This discrepancy may be caused by the differences in the studied population, such as a high proportion of patients in remission and with the disease localized to the colon, since the disease localization may influence local and serum levels of MMPs [61]. Neither glucocorticoids nor TNF-α blockers influenced MMP-9 levels similarly as found in pediatric IBD patients by others [61].

Low serum TGF-β1 was the strongest factor associated with the active disease (relapse) as compared to quiescent disease (remission) and together with other factors (TFF-3, MMP-9 and LBP) was capable of distinguishing between these two conditions with high accuracy (AUC = 0.909). TGF-β is important cytokine for the maintenance of intestinal homeostasis through its immunoregulatory functions, gut barrier support and wound healing [62,63,64]. However, by promoting collagen III production by myofibroblasts it is partially responsible for typical CD complications, such as intestinal fibrosis, fistulae and strictures [65]. While our findings of elevated anti-inflammatory TGF-β1 in remission as compared to the active disease may be counterintuitive, one previous study already described similar findings in pediatric IBD [66]. This suggests that high TGF-β1 in remission may reflect the organism’s successful effort to dampen the inflammation during IBD, which makes TGF-β1 an interesting potential candidate for relapse prediction.

Gut barrier failure and immune response to gut commensal microbiota are both hallmarks of IBD. The barrier failure leads to exposure of the immune cells in the gut mucosa to bacterial antigens, thus anti-bacterial immune response may serve as an indirect marker of chronic gut barrier failure. In order to measure which bacteria are targeted most in IBD as well as in HC, we selected 10 bacteria covering typical gut bacteria found in healthy Czech subjects [41]. We found that there are generally no differences between IBD patients and healthy controls except for patients suffering from CD that have generally lower antibody response against gut commensals. While the CD patients did not significantly differ from HC in IgM and IgG response, we found significantly decreased IgA response to *Faecalibacterium* and *Bacteroides* in CD patients compared with HC. In fact, 23% of UC patients and 40% of CD patients have undetectable IgA response to *Faecalibacterium*. In the normal healthy gut *F. prausnitzii* accounts for more than 5% of the total bacterial microbiota and is one of the most abundant commensal species [41,67], but it is markedly under-represented in the gut of patients with CD [68,69]. In fact, its low abundance on ileal mucosa or in feces predicts relapse in CD patients [69,70]. *F. prausnitzii* is able to produce not only anti-inflammatory molecules such as butyrate, but it can modulate the host’s immune response with a specific anti-inflammatory protein [71]. Our data suggest that the absence of *F. prausnitzii* during the inflammation in CD leads to a decrease in antibody response. We may speculate that significant decrease in IgA response to *Bacteroides* and non-significant decrease in *Blautia* and *Roseburia* may be caused by similar mechanisms, because many butyrate-producing bacteria, including *Blautia faecis*, *Roseburia inulinivorans* and *Bacteroides uniformis*, are significantly reduced in the gut of CD patients as compared to healthy controls [72]. There is, however, limitation to this assay, because many antigens are shared among species of a particular bacterial genus, while changes in abundances and or biological activities may be specific to a particular species or even isolate [73].

T cell response to microbiota plays an important role in IBD pathogenesis and commensal gut bacteria provide antigenic stimulation that can activate pathogenic T cells and lead to chronic intestinal inflammation [74]. Specific polarization of these cells is linked to the particular type of IBD, with Th1 and Th17 T cells are associated with CD and Th2 T cells are often associated with UC [75,76]. However, gut microbiota-specific cells are normally property of the memory T helper repertoire of PBMCs and do not necessarily indicate interaction between immune cells and the gut commensal microbiota. Nevertheless, the cytokine profile of these cells is changed during the intestinal inflammation [43]. Therefore, we used multi-color flow cytometry to analyze how differences in their cytokine profiles reflect different forms and states of IBD. We found that T helper cells from healthy subjects quickly up-regulate CD154 when stimulated with antigens from *Blautia*, *Roseburia*, *Prevotella*, *Lactobacillus* and *Escherichia* and that this spectrum of reactivity is generally broadened in patients with IBD. In our experiments, we focused on the response to bacteria found in the Czech population, so the reactivity to these particular microbes may not be universal worldwide.

When we focused on individual cytokines, we found only minor differences among the individual groups of IBD patients and HC, with a general increase in cytokine-producing memory T cells in CD group. Despite this trend, there were no significant differences in IFN-γ^+^CD154^+^CD4^+^ T cells. Antigens from *Roseburia* had significant impact on memory T cells from PSC-IBD and CD as compared to HC and antigens from *Eubacterium, Faecalibacterium* and *Bacteroides* had significantly different impacts on memory T cells from PSC-IBD and CD as compared to HC. This suggests that while specific IgA response to *Roseburia, Faecalibacterium* and *Bacteroides* is decreased in patients with CD, their memory T cells react more strongly to these particular microbes. Unfortunately, in all bacteria lysate-stimulated samples the numbers of IL-4^+^CD154^+^CD4^+^ T cells were so low, that we were not able to perform reliable analysis, and had to exclude it from analyses. Interestingly, we found a significantly higher proportion of IL-17^+^CD154^+^ cells in CD patients and a similar trend in UC patients, but not in PSC-IBD group, when stimulated with the super-antigen SEB. We did not observe any other differences in super-antigen-stimulated samples. This suggests that, unlike circulating memory T cells from patients with CD (and to a lesser degree in patients with UC), those from patients with PSC-IBD do not have an increased capacity to form Th17 cells.

Decreasing the inflammatory response by steroidal and non-steroidal anti-inflammatory drugs, biologicals and other immunomodulators is a cornerstone of IBD therapy and each type and severity of IBD requires an individual therapeutic approach. For example, anti-TNF-α is only rarely used in PSC-IBD patients and more severe cases of UC and CD are often treated with combination of drugs. This individual approach was present in our cohort as well, with 5-ASA used more in PSC-IBD patients than in UC patients and anti-TNF-α treatment is used only in UC and CD patients. The specificities of IBD pharmacotherapy limit the generalization of this study, but it does not make it irrelevant for clinical use, because the patients will be treated differently in the future. Therefore, we analyzed the effect of each therapeutic intervention in all patients with intestinal inflammation to find the therapy-specific biomarkers and to gauge the impact of the pharmacotherapy on the analyzed biomarkers. The effect was generally mild, with only a few notable exceptions. The significant increase in MMP-14 in patients treated with 5-ASA could be responsible for its general increase in IBD patients, because 82% of them were treated with 5-ASA. A similar effect could be partially responsible for the higher levels of OPG in PSC-IBD as compared to UC, because anti-TNF-α was not used in any PSC-IBD patient. Moreover, we found an increase in IFN-γ production from *Roseburia*- or *Escherichia*-treated CD154^+^CD4^+^ T cells and increased TNF-α production from *Escherichia* treated CD154^+^CD4^+^ T cells in patients treated with anti-TNF-α. This may represent a biological background for rebound phenomenon when TNF-α blockers are excluded from the system and unchecked biological feedback increases the pro-inflammatory response. The opposite trend for Azathioprine, which acts directly on the cells, may reflect the biological background for the recently published meta-analysis finding the significant decrease in relapse rate when anti-TNF-α is discontinued under the screen of immune-modulators [77].

## 5. Conclusions

In this study we established the panels of biomarkers reflecting the specificities of pathogenesis of the different forms of IBD that may represent interesting future biomarkers. While the MMP system seems to be the strongest discriminator between healthy subjects and IBD patients, we identified markers reflecting colitis activity and anti-TNF-α treatment. Furthermore, we performed comprehensive screening of humoral and cellular adaptive immune response against gut commensal bacteria, finding several clear differences between healthy subjects and IBD patients, most notably CD. In general, these consisted of decreased IgA response to *Faecalibacterium* and *Bacteroides* with an increased T cell response to similar bacteria. These results stress the importance of gut barrier function and immune response to commensal bacteria in IBD pathogenesis and clearly show that PSC-IBD, UC and CD each represent a distinct form of IBD.

## Figures and Tables

**Table 1:** Clinical characteristics of the study participants. CD: Crohn’s disease; HC: healthy control; PSC: primary sclerosing cholangitis; UC: ulcerative colitis.

	HC *n* = 28	PSC *n* = 47	UC *n* = 52	CD *n* = 20
Age (mean ±SD; years)	42.5 ± 10.5	38.0 ± 11.6	39.7 ± 9.8	33.5 ± 7.8
Sex (% of males)	53.6; 15/13	74.5; 35/12	53.9; 28/24	45.0; 9/11
Activity (% of active)	0.0	14.0	26.9	20.0
Extent of intestinal inflammation				
none (%; n)	100.0; 28	12.8; 6	0.0; 0	0.0; 0
partial (%; n)	0.0; 0	10.6; 5	38.4; 20	45.0; 9
pancolitis (%; n)	0.0; 0	72.3; 34	61.5; 32	50.0; 10
Therapy				
Mesalazine (5-ASA) (%; n)	0.0; 0	70.2; 33	92.3; 48	85.0; 17
Glucocorticoids (%; n)	0.0; 0	38.3; 18	21.2; 11	15.0; 3
Azathioprine (AZA) (%; n)	0.0; 0	31.9; 15	40.4; 21	35.0; 7
Anti-TNF-α (%; n)	0.0; 0	0.0; 0	38.5; 20	45.0; 9
*E. coli* Nissle 1917 (%; n)	0.0; 0	8.5; 4	23.1; 12	20.0; 4

**Table 2:** List of biomarkers quantified in sera of inflammatory bowel disease (IBD) patients and healthy subjects.

Biomarker	Abbreviation	Manufacturer	Cat. No
Endocrine-Gland-derived Vascular Endothelial Growth Factor *	EG-VEGF	R&D systems	DY1209
Interleukin-8 receptor, alpha *	CXCR1/IL8RA	LifeSpan BioScience	LS-F11255
Osteoprotegerin	OPG	R&D systems	DY805
Tomoregulin 1	TMEFF1	LifeSpan BioScience	LS-F52730
Insulin-like Growth Factor 2 *	IGF2	R&D systems	DY292
Transforming Growth Factor-β1 *	TGF-β1	R&D systems	DY240
TROY protein	TNFRSF19	LifeSpan BioScience	LS-F39966
Roundabout Guidance Receptor 4 *	ROBO4	RayBiotech	ELH-ROBO4
Matrix Metalloproteinase 9	MMP-9	R&D systems	DY911
Matrix Metalloproteinase 14	MMP-14	R&D systems	DY918
Tissue Inhibitor of Metalloproteinases 1	TIMP-1	R&D systems	DY970
Mannan-Binding Lectin	MBL	R&D systems	DY2307
Soluble CD14	CD14	R&D systems	DY383
Lipopolysaccharide-Binding Protein	LBP	R&D systems	DY870
Trefoil Factor 3	TFF-3	R&D systems	DY4407
Endotoxin-Core Antibody IgM	EndoCab	MyBiosource	MBS9352896
Serum Amyloid A	SAA	HyCult Biotech	HK333
Pre-haptoglobin 2	Zonulin	MyBiosource	MBS2880564
D-amino-acid oxidase	DAAO	MyBiosource	MBS2886321
Intestinal Fatty Acid-Binding Protein	I-FABP	HyCult Biotech	HK406
Liver Fatty Acid-Binding Protein	L-FABP	HyCult Biotech	HK404

* Identified by the array.

## Supplementary Materials

The following are available online at https://www.mdpi.com/2073-4409/8/7/719/s1. Table S1: Bacteria and cultivation conditions. Figure S1: Significant differences in serum biomarkers between PSC-IBD, UC and CD patients and healthy controls as analyzed by the Kruskal–Wallis test with Dunn’s multiple comparison test. Different letters indicate statistically significant differences. Figure S2: Serum biomarkers do not describe the extent of colitis well. (A) Heat map and cluster analysis of the chosen proteins. (B) Composite ROC curve analysis for the biomarkers discriminating between partial and total colonic inflammation using the best model found by regression analysis. PSC-IBD (*n* = 29), UC (*n* = 34), CD (*n* = 19). Figure S3: Only a few serum biomarkers are influenced by IBD treatment, but their discriminating power is generally low. (A) Heat map and cluster analysis of the serum biomarkers. (B) Relative importance of each cytokine for the AUC increment within the best model found by regression analysis and quantitative plot of the strongest discriminating factor analyzed by the Mann–Whitney test. * *p* < 0.05, ** *p* < 0.01; other = anti-α4β7 PSC-IBD (*n* = 31), UC (*n* = 34), CD (*n* = 20). Figure S4: Serum antimicrobial antibodies are influenced by IBD treatment only to a limited degree. (A) Heat map and cluster analysis of the serum biomarkers. (B) Relative importance of each anti-microbial antibody for the AUC increment within the best model found by regression analysis and quantitative plot of the strongest discriminating factor analyzed by Mann Whitney test. * *p* < 0.05; other = anti-α4β7; PSC-IBD (*n* = 32), UC (*n* = 37), CD (*n* = 20). Figure S5: Anti-TNF-α treatment influences cellular antimicrobial response in IBD patients. (A) Heat map and cluster analysis of the serum biomarkers. (B) Quantitative plot of the strongest discriminating factors selected by correlation analysis as analyzed by the Mann–Whitney test. *** *p* < 0.001. PSC-IBD (*n* = 8), UC (*n* = 14), CD (*n* = 17), AZA treated (*n* = 13), AZA non-treated (*n* = 26), anti-TNF-α treated (*n* = 10), anti-TNF-α non-treated (*n* = 27). Figure S6: Response of memory T helper cells has a specific profile for each healthy control (*n* = 14) and PSC-IBD (*n* = 9), UC (*n* = 12) and CD (*n* = 13) patient as analyzed by non-parametric paired Friedman test with Dunn’s multiple comparison test. * *p* < 0.05, ** *p* < 0.01, *** *p* < 0.001. Figure S7: Gating strategy for flow cytometry using SEB-stimulated PBMCs
