# Supplementary material for: Inflammatory Bowel Disease Types Differ in Markers of Inflammation, Gut Barrier and in Specific Anti-Bacterial Response
Source: Cells. 2019 Jul 13;8(7):719. doi: 10.3390/cells8070719 (PMC6678638; doi:10.3390/cells8070719)
Supplement: Supplementary file 1 [file cells-08-00719-s001.pdf]

cells

Supplementary Materials:

Bacterium	Cultivation medium	Cultivation condition
Lactobacillus plantarum CCDM 182	MRS Broth for Lactobacilli (ATCC Medium No. 416)	anaerobic
Bifidobacterium adolescentis CCUG 18363	DSMZ medium No. 58. for Bifidobacteria	anaerobic
Blautia coccoides	Modified chopped meat medium (ATCC Medium No. 1490)	anaerobic
Roseburia intestinalis L1-82	Keister's Modified TYI-S-33 (ATCC Medium No. 2695)	anaerobic
Eubacterium rectale ATCC 33656	Chopped meat carbohydrates with rumen fluid (ATCC Medium No 1703)	anaerobic
Faecalibacterium prausnitzii A2-165	Modified YCFA medium (DSMZ medium No. 1611)	anaerobic
Ruminococcus flavefaciens DSM 25089	Medium for anerobes with 0.1% cellobiose (ATCC Medium No. 1365 E)	anaerobic
Bacteroides thetaiotaomicron VPI 5482	Modified chopped meat medium (ATCC Medium No. 1490)	anaerobic
Prevotella ruminicola M384	Chopped meat carbohydrates with rumen fluid (ATCC Medium No 1703)	anaerobic
Escherichia coli K6	Luria-Bertani broth (Merck, L3022)	aerobic

Table S1: Bacteria and cultivation conditions

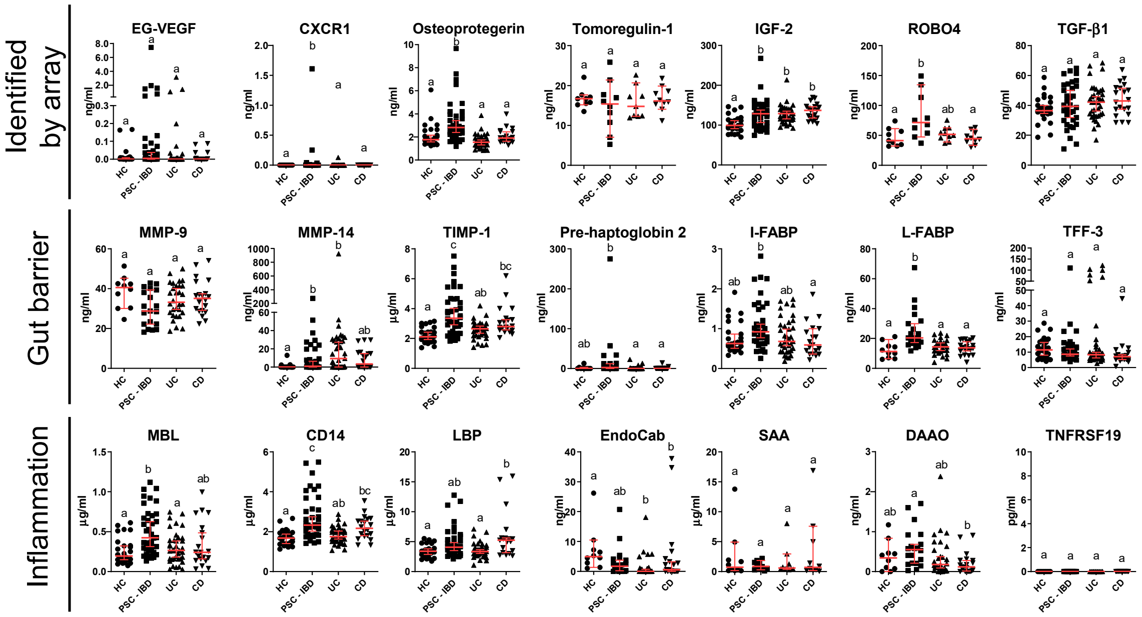

Figure S1: Significant differences in serum biomarkers between PSC-IBD, UC and CD patients and healthy controls as analyzed by Kruskal-Wallis test with Dunn's multiple comparison test. Different letters indicate statistical significant differences.

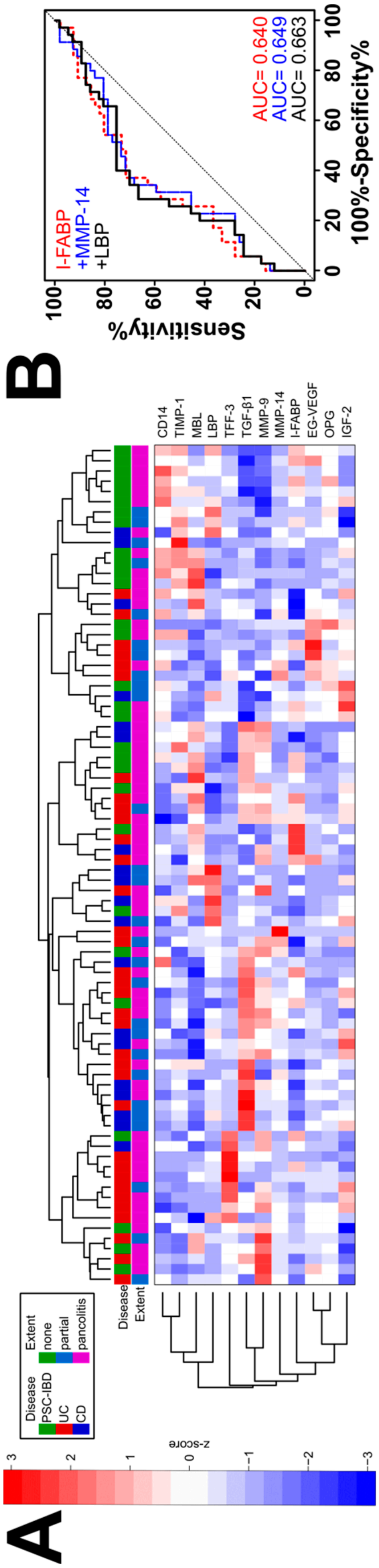

Figure S2: Serum biomarkers do not describe the extent of colitis well. (A) Heat map and cluster analysis of the chosen proteins. (B) Composite ROC curve analysis for the biomarkers discriminating between partial and total colonic inflammation using the best model found by regression analysis. PSC-IBD (n=29), UC (n=34), CD (n=19).

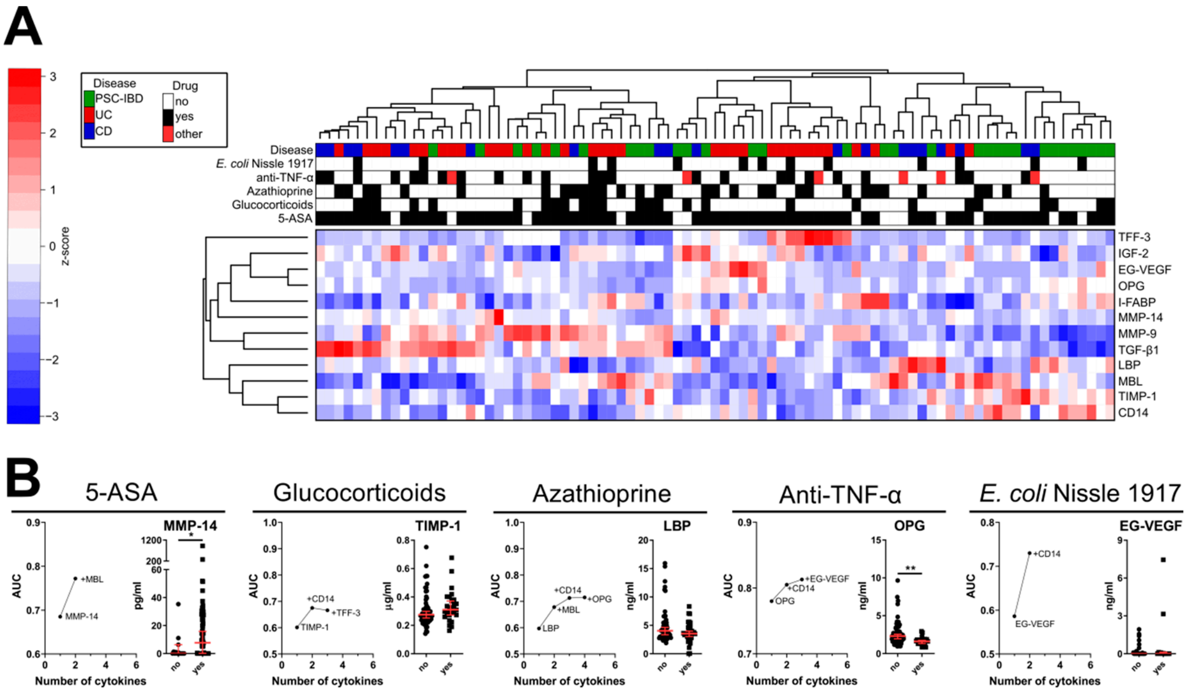

Figure S3: Only few serum biomarkers are influenced by IBD treatment, but their discriminating power is generally low. (A) Heat map and cluster analysis of the serum biomarkers. (B) Relative importance of each cytokine for the AUC increment within the best model found by regression analysis and quantitative plot of the strongest discriminating factor analyzed by Mann Whitney test. * $p<0.05$, ** $p<0.01$; other = anti-$\alpha 4\beta 7$ PSC-IBD (n=31), UC (n=34), CD (n=20).

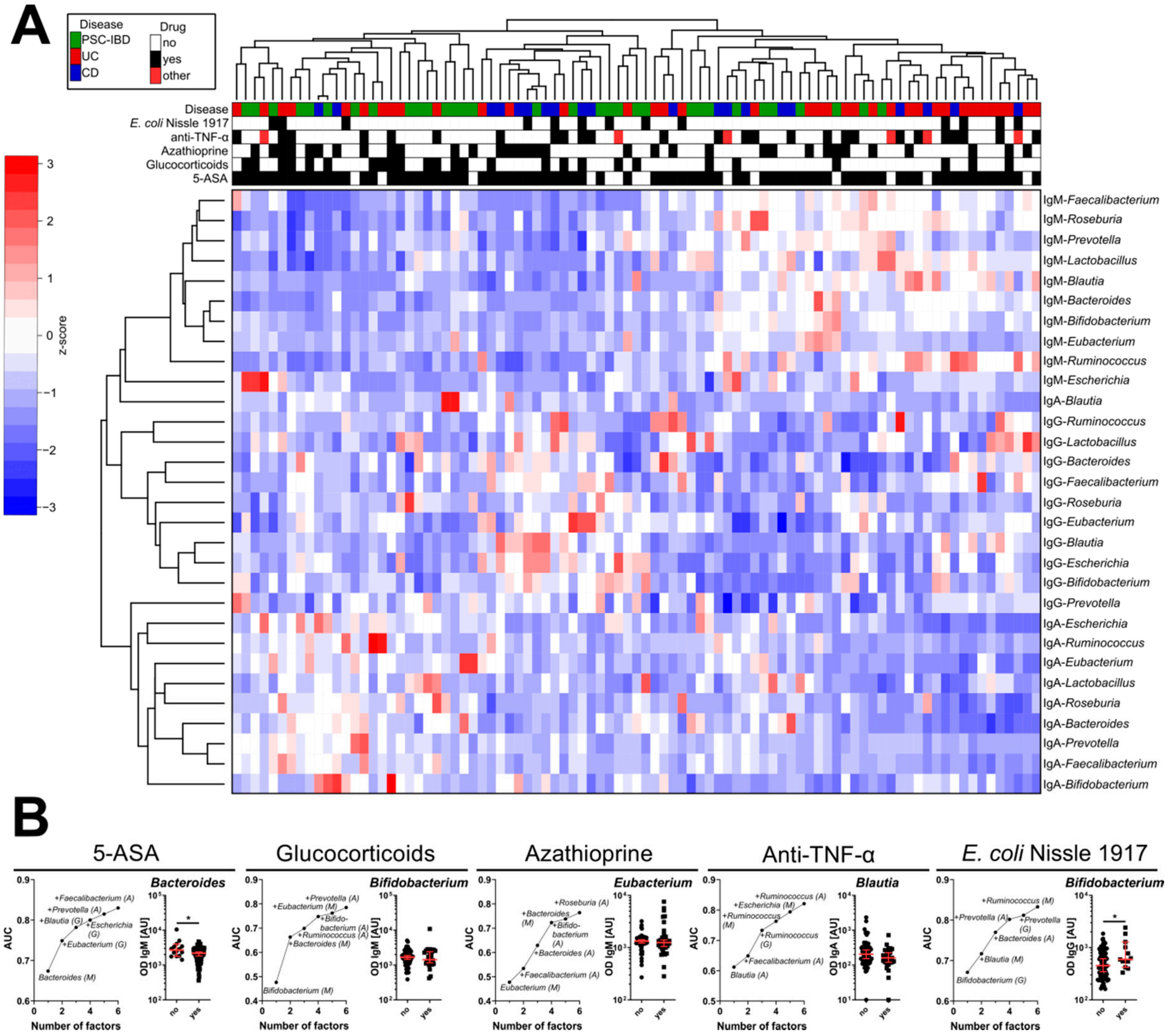

Figure S4: Serum antimicrobial antibodies are influenced by IBD treatment only to a limited degree. (A) Heat map and cluster analysis of the serum biomarkers. (B) Relative importance of each anti-microbial antibody for the AUC increment within the best model found by regression analysis and quantitative plot of the strongest discriminating factor analyzed by Mann Whitney test. * $p<0.05$; other = anti-$\alpha 4\beta 7$; PSC-IBD (n=32), UC (n=37), CD (n=20).

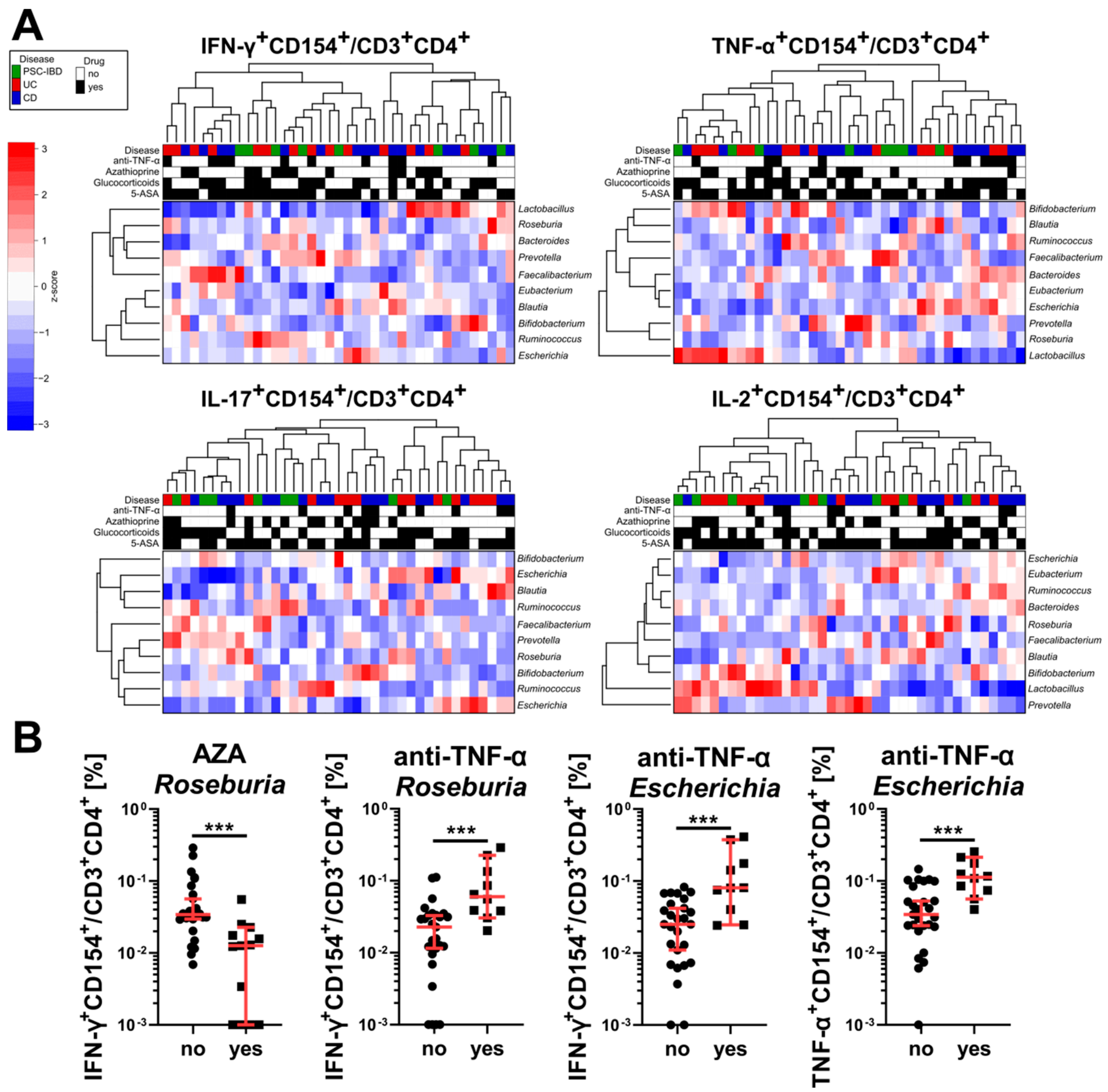

Figure S5: Anti-TNF-α treatment influences cellular antimicrobial response in IBD patients. (A) Heat map and cluster analysis of the serum biomarkers. (B) Quantitative plot of the strongest discriminating factors selected by correlation analysis as analyzed by Mann Whitney test. *** p<0.001. PSC-IBD (n=8), UC (n=14), CD (n=17), AZA treated (n=13), AZA non-treated (n=26), anti-TNF-α treated (n=10), anti-TNF-α non-treated (n=27)

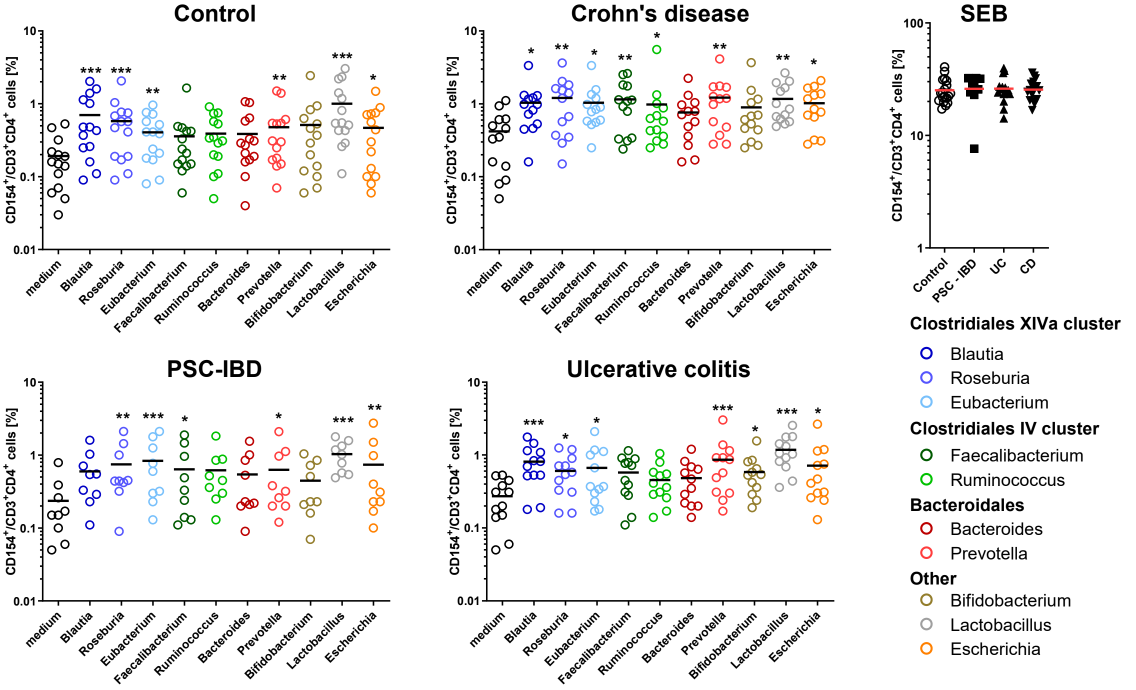

Figure S6: Response of memory T helper cells has a specific profile for each healthy controls (n=14) and PSC-IBD (n=9), UC (n=12) and CD (n=13) patients as analyzed by non-parametric paired Friedman test with Dunn's multiple comparison test. * $p<0.05$, ** $p<0.01$, *** $p<0.001$

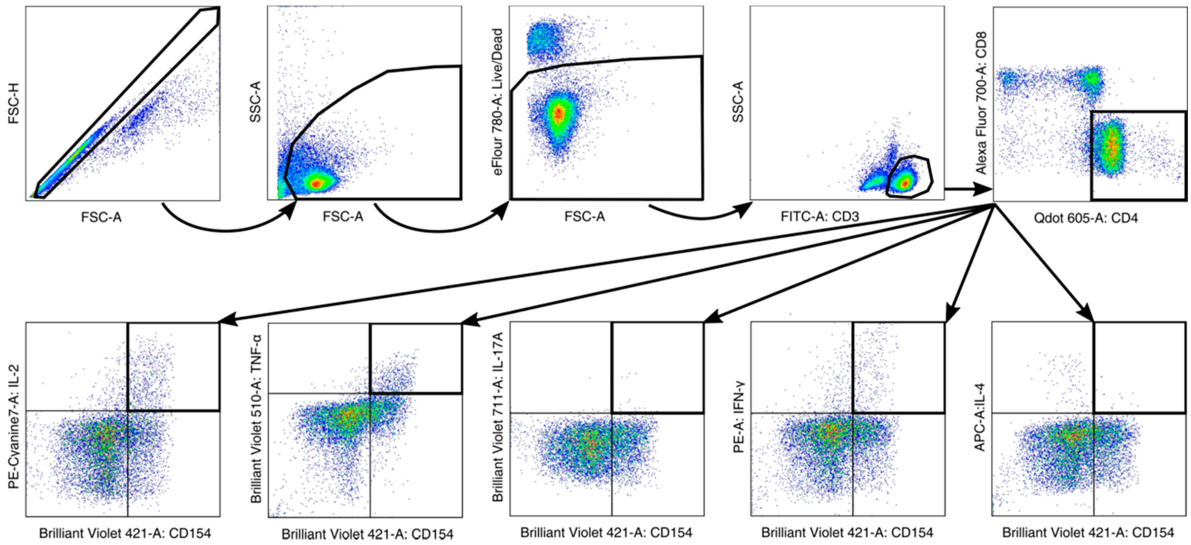

Figure S7: Gating strategy for flow cytometry using SEB-stimulated PBMCs
